# Robotic-assisted versus conventional total knee arthroplasty: a systematic review and meta-analysis of randomized controlled trials

**DOI:** 10.1007/s00590-023-03798-2

**Published:** 2023-12-22

**Authors:** Reda Alrajeb, Mohammed Zarti, Zakaria Shuia, Osama Alzobi, Ghalib Ahmed, Aissam Elmhiregh

**Affiliations:** 1 Almowasafat Hospital, Tripoli, Libya; 2 Department of Orthopedic Surgery, Surgical Specialty Center, Hamad Medical Corporation, Doha, Qatar

**Keywords:** Total knee arthroplasty, Robotic arthroplasty, Jig-based arthroplasty, Conventional knee arthroplasty

## Abstract

**Objectives:**

Robotic knee arthroplasty procedures have emerged as a new trend, garnering attention from orthopedic surgeons globally. It has been hypothesized that the use of robotics enhances the accuracy of prosthesis positioning and alignment restoration. The objective of this study was to provide a high-level, evidence-based comparison between robotic total knee replacements and conventional methods, focusing on radiological and functional outcomes.

**Methods:**

We searched five databases from their inception until June 1, 2022, specifically targeting randomized controlled trials (RCTs) that compared the outcomes of robotic and conventional total knee replacements. We were interested in outcomes such as knee range of motion, clinical and function knee society scores, the Western Ontario and McMaster University score (WOMAC), the Hospital of Special Surgery score, complications, and radiological alignment. This review was carried out in accordance with the Preferred Reporting Items for Systematic Reviews and Meta-Analyzes guidelines. We assessed the risk of bias using the revised Cochrane risk-of-bias tool for randomized trials (RoB 2).

**Results:**

Our search returned seven RCTs suitable for our analysis, which included a total of 1942 knees; 974 of these knees were implanted using robotic arms while the remaining 968 utilized jig-based knee systems. Our findings indicated that robotic knees had significantly better post-operative anatomical (OR − 0.82; 95% CI, − 1.027 to − 0.58, *p* value < 0.00001) and mechanical restoration (OR − 0.95; 95% CI, − 1.49 to − 0.41, *p* value < 0.0006). While knee range of motion (OR − 2.23; 95% CI − 4.89–0.43, *p* value 0.1) and femoral prosthesis position (OR − 0.98; 95% CI, − 2.03–0.08, *p* value 0.07) also favored robotic knees, these differences did not reach statistical significance. Both clinical and functional outcomes, as well as the rate of complications, were found to be statistically similar between the groups undergoing robotic and traditional knee replacement surgeries.

**Conclusion:**

This meta-analysis indicates that robotic total knee replacements offer superior post-operative anatomical and mechanical alignment compared to conventional total knee replacements. Despite this, clinical and functional outcomes, as well as complication rates, were similar between the two. These findings should be considered in light of potential confounding factors. More randomized controlled trials with the latest robotic systems are needed to confirm any superior functional and clinical outcomes from robotic-assisted surgeries.

**Level of evidence:**

I.

## Introduction

Over the last few decades, total knee replacement has experienced significant growth as an orthopedic procedure. From 2017 to 2019 alone, the UK recorded 312,167 primary knee replacements, constituting 24% of the national joint registry [1]. This surge can largely be linked to the increasing prevalence and economic impact of osteoarthritis among different populations [2].

Despite the proven effectiveness and replicability of traditional knee arthroplasty methods, paired with advancements in prosthesis technology, a considerable number of patients are still unhappy with their knee replacements for various known and undetermined reasons [3, 4]. Factors such as soft tissue balance and implant placement have been identified to influence patient satisfaction with the procedure [5, 6]. Though precise component alignment is often achievable and replicable in today's conventional total knee arthroplasty (CTKA), it can be challenging and intricate to obtain, particularly in more complex cases [7].

The development of robotic total knee arthroplasty (RTKA) was aimed at eliminating potential inaccuracies in implant positioning and alignment, thus reducing patient dissatisfaction. The first-ever RTKA was performed in the UK as early as 1988, utilizing the Acrobat system [8]. Given that most robotic systems employed in knee arthroplasty use 3-dimensional imaging, it is suggested that these robotic procedures offer higher precision in positioning and balancing. Some reports even suggest that they outperform traditional jig-based methods in clinical settings [9, 10]. However, this claim has not yet been conclusively validated [11].

Over the past decades, various robotic arms have been developed and incorporated into medical practice. These include systems such as ROBODOC by Curexo Technology in Fremont, CA [12], Mako by Stryker in Mahwah, NJ [13], CASPAR by URS Ortho GMBH and Co in Rastatt, Germany [14], and NAVIO by Smith and Nephew in Memphis, TN [15].

The objective of this meta-analysis was to provide the most robust evidence currently available that compares these two techniques. To the best of our knowledge, this is the only meta-analysis that draws upon randomized controlled trials to compare robotic total knee arthroplasty with its conventional equivalent in the existing literature.

Our hypothesis was that there would be no significant difference between the two groups concerning functional results, knee range of motion and rates of complications.

## Materials and methods

This meta-analysis was conducted in line with the guidelines of the Preferred Reporting Items for Systematic Reviews and Meta-Analyzes (PRISMA), utilizing both a PRISMA checklist and algorithm [16].

### Search strategy

PubMed/Medline, CINAHL, Cochrane, Embase, and Google Scholar databases were systematically searched from inception until June 1, 2022, to identify articles in peer-reviewed journals. The search was performed using the following keywords and their derivatives: “Knee arthroplasty,” “Knee replacement,” “Joint replacement,” “Total knee,” “Robotic-assisted,” “Conventional,” “Robotic-arm,” “Randomized,” and “RCT.” Two authors independently sifted through search results, assessing them against the eligibility criteria based on their titles and/or abstracts. Any disagreements were addressed in a resolution meeting with a third senior author. A comprehensive review of the full-text articles that met the eligibility criteria was conducted, and references from these articles were manually checked to guarantee the inclusion of all pertinent studies.

### Eligibility criteria

#### Inclusion criteria

1. All original comparative level I of evidence randomized controlled trials (RCTs) reporting primary TKA indicated in robotic total knee arthroplasty with its conventional equivalent.
2. The primary indication for TKA is primary osteoarthritis.
3. English full-text manuscript with available data.
4. RCTs that published clear outcome measures with attached data presented as or can be transferred to mean and standard deviation values.

#### Exclusion criteria

1. Studies involving patients with inflammatory arthritis or post-traumatic arthritis.
2. Non-comparative or not reporting outcomes.
3. Review articles, cross-sectional, case series and reports.
4. Preclinical or animal studies.

### Data collection process and data items

A pre-designed data collection sheet in Microsoft Excel was utilized by two independent reviewers to extract data. The collected data items comprised: surname of the first author, year of study, age, gender, patient count, prosthesis type, robotic system used, points of follow-up, revision rates for any cause, complications encountered.

### Outcomes of interest

The study evaluated several outcomes of interest, including scores for knee society pain and function, the Western Ontario and McMaster University score (WOMAC), the Hospital of Special Surgery score (HSS), knee range of motion, and alignment parameters. Additionally, we were successful in obtaining data related to sagittal and coronal knee alignments, which included the anterior–posterior (AP) tibial angle (varus–valgus), tibial slope angle, femoral flexion angle, and tibiofemoral angle.

### Qualitative assessment (risk of bias)

The qualitative analysis was conducted using the revised Cochrane risk-of-bias tool for randomized trials (RoB 2) [17]. This tool evaluates five key areas: randomization, adherence to intended treatments, missing outcomes, measurement bias, and reporting bias. Two authors independently assessed each study using the RoB 2, and the final evaluation of each study was reviewed in conjunction with the senior author to arrive at a mutual agreement.

### Quantitative analysis

RevMan V.5.0.18.33 (The Cochrane Collaboration, Copenhagen, Denmark) was used to perform the meta-analysis. Mean and standard variations were extracted to present continuous variables. Dichotomous variables were analyzed by Relative risk (RR) with 95% CI. Heterogeneity was measured using *I*^2^, and results were considered statistically significant at *p* < 0.05.

## Results

### Study selection

**Fig. 1:**
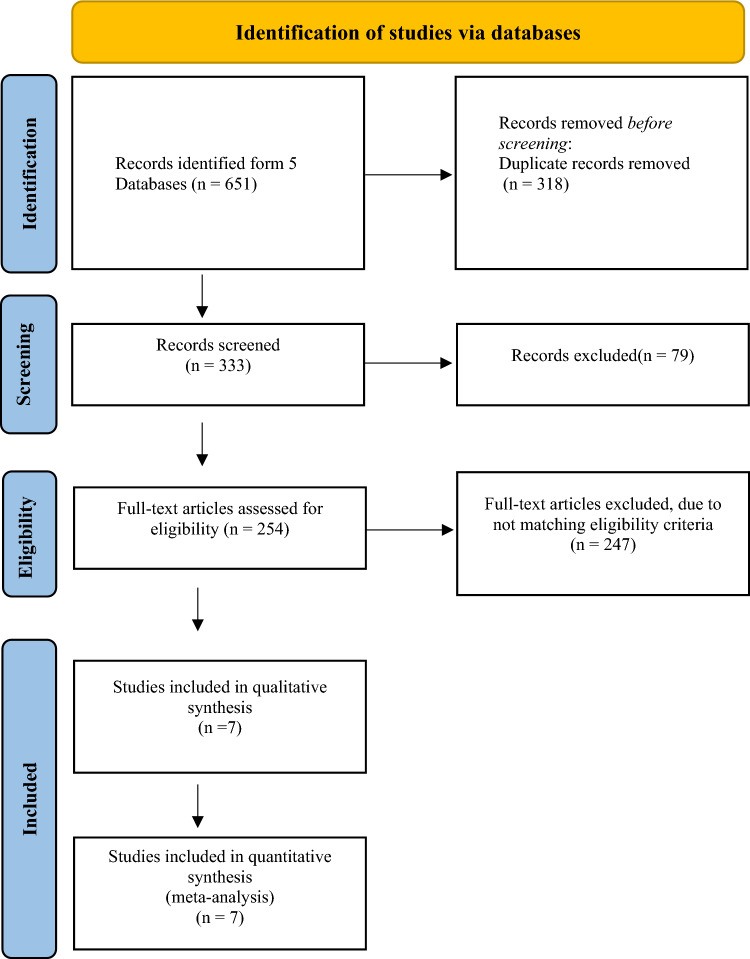
Search strategy flowchart

The initial search resulted in 651 articles; among these, 318 were duplicates and were removed both manually and electronically. The remaining articles were screened based on title and abstract, resulting in the exclusion of 79. The full text of the 333 remaining articles was evaluated against the inclusion criteria. Ultimately, seven studies fulfilled the eligibility criteria and were incorporated into both the qualitative and quantitative synthesis [18–24]. In instances where studies provided details on approximately the same cohort at two different time points, only results from the later study were included [19, 25]. The PRISMA flowchart detailing this process is displayed in Fig. 1.

### Characteristics of the included studies

In our final qualitative and quantitative analysis, seven randomized controlled trials met our inclusion criteria and were thus eligible for our analysis. The study included 1942 knees; of these, 974 were implanted using robotic arms and the remaining 968 were conventionally implanted. Although different prosthesis designs and brands were used in total, each included study compared outcomes using the same knee brand and design. The studies included in this analysis did not contain data on cementless TKAs. All TKAs in the included RCTs were cemented. In terms of robotic systems, five papers [18–22] utilized the ROBODOC system (Integrated Surgical Systems, Sacramento, CA) for the robotically assisted knee group, while the other two studies [23, 24] used the NAVIO® system (Smith and Nephew, Andover, TX, USA).

All the trials included in our study matched their study groups in terms of participant age and gender. However, there was a variation in the follow-up periods, so we reported our results at the final follow-up. Two studies [23, 24] provided post-operative radiological outcomes without specifying a follow-up interval, which is understandable given that their outcome measures were solely radiological assessments. We also examined common outcomes between the studies. Despite the variation in follow-up periods, we analyzed the data based on the final patient visits (Table 1).

**Table 1:** Characteristics of the included studies

Study	LoE	Number of knees	RTKA	CTKA	Follow up (month)	Type of prosthesis	Robot system
Park [20]	I	62	32	30	47.17	Zimmer LPS	ROBODOC
Song [21]	I	60	30	30	12	NexGen CR prosthesis (Zimmer, Warsaw, Indiana)	ROBODOC
Song [22]	I	100	50	50	36	NexGen CR prosthesis (Zimmer, Warsaw, Indiana)	ROBODOC
Liow [19]	I	60	31	29	24	NexGen LPS-Flex; Zimmer, Inc, Warsaw, IN, USA	ROBODOC
Kim [18]	I	1448	724	724	120	Duracon® posterior cruciate-substituting total knee prosthesis (Stryker Orthopedics, Mahawh, NJ, USA)	ROBODOC
Vaidya [24]	I	60	32	28	0	posterior-stabilized prosthesis (Anthem, Smith and Nephew Inc.)	Navio
Thiengwittayaporn [23]	I	152	75	77	0	(Legion® PS Total Knee System, Smith and Nephew, Memphis, TN, USA)	Navio

*LoE* level of evidence, *RTKA* robotic total knee arthroplasty, *CTKA* conventional total knee arthroplasty

### Quality assessment

Three studies demonstrated a low risk of bias, while five studies exhibited some concern for bias. Notably, none of the included studies showed a high risk of bias. All studies maintained their groups according to the original randomization, and no study experienced a high dropout rate or failed to report outcomes. A graphic representation of the qualitative assessment can be found in Table 2.

**Table 2:**
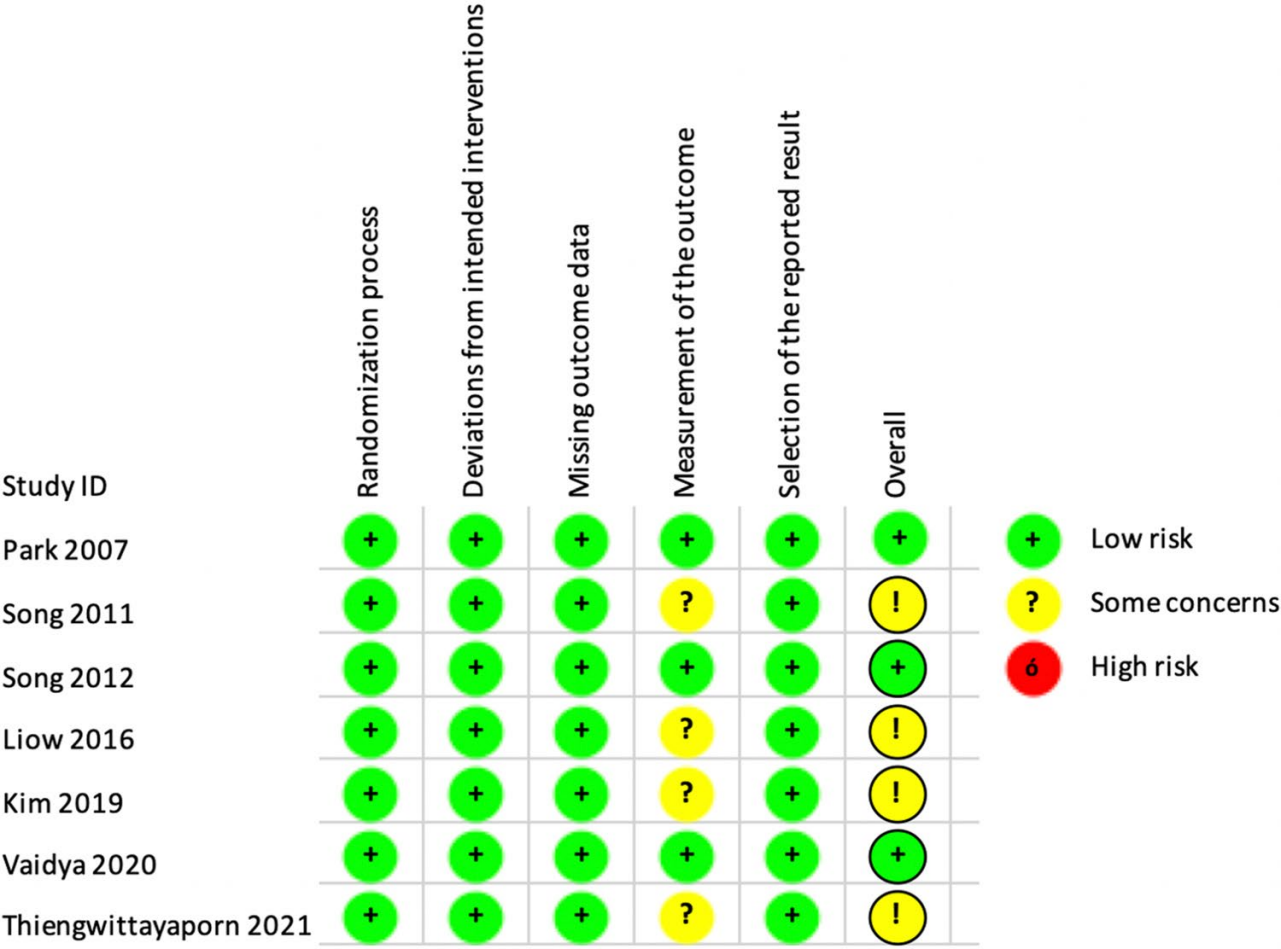
Risk of bias in individual studies

### Clinical knee society score (KSS clinical)

**Fig. 2:**
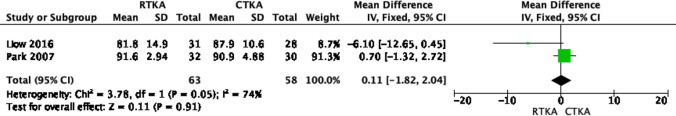
Forest plot of clinical knee society score at the final follow up between RTKA and CTKA, CI confidence interval

The clinical knee society score was documented in two studies [19, 20]. These studies reported their outcomes at varying follow-up periods, ranging from 24 to 47 months. We reported our meta-analysis outcome at the final follow-up. None of the included studies, nor our fixed model analysis, reported a statistically significant difference. Figure 2 presents the forest plot of the clinical knee society score, revealing no statistical difference between the two groups (OR 0.11; 95% CI − 1.82–2.04, *p* value 0.91) and high heterogeneity across the results (*I*^2^ = 74%).

### Functional knee society score (KSS functional)

**Fig. 3:**
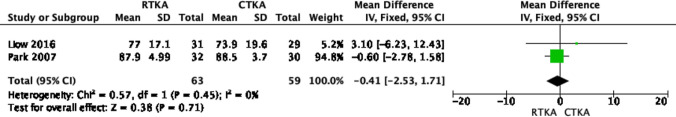
Forest plot of functional knee society score at the final follow up between RTKA and CTKA, CI confidence interval

The functional knee society score was outlined in two studies [19, 20]. Due to variations in follow-up periods among the included studies, we reported the outcome at the final follow-up. Figure 3 shows the forest plot of the clinical knee society score, which demonstrates no statistical difference between the two groups (OR − 0.41; 95% CI − 2.53, 1.71, *p* value 0.71). The results also displayed low heterogeneity (*I*^2^ = 0%).

### Hospital of special surgery score (HSS)

**Fig. 4:**
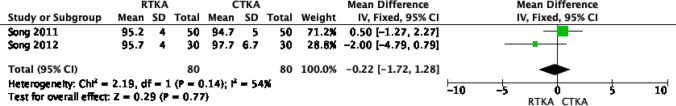
Forest plot of hospital of special surgery score at the final follow up between RTKA and CTKA, CI confidence interval

Two studies [21, 22] reported the HSS score at 12 and 36 months, respectively. Our fixed model analysis, conducted at the final follow-up, did not reveal any differences between the two groups. This is represented in Fig. 4 (OR − 0.22; 95% CI − 1.72, 1.28, *p* value 0.14).

### The Western Ontario and McMaster Universities Osteoarthritis Index (WOMAC) functional score

**Fig. 5:**
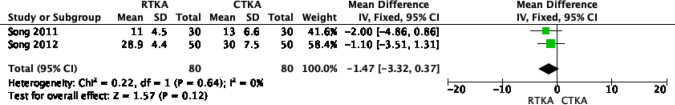
Forest plot of WOMAC score at the final follow up between RTKA and CTKA, CI confidence interval

The WOMAC score was examined in two papers [21, 22], with neither reporting any differences between RTKA and CTKA at 12 and 36 months, respectively. Our fixed model analysis, conducted at the final follow-up, also showed no significant differences. These findings are illustrated in Fig. 5 (OR − 1.47; 95% CI − 3.32–0.37; *p* value 0.12), with the results demonstrating low heterogeneity (*I*^2^ = 0%).

### Knee range of motion

**Fig. 6:**
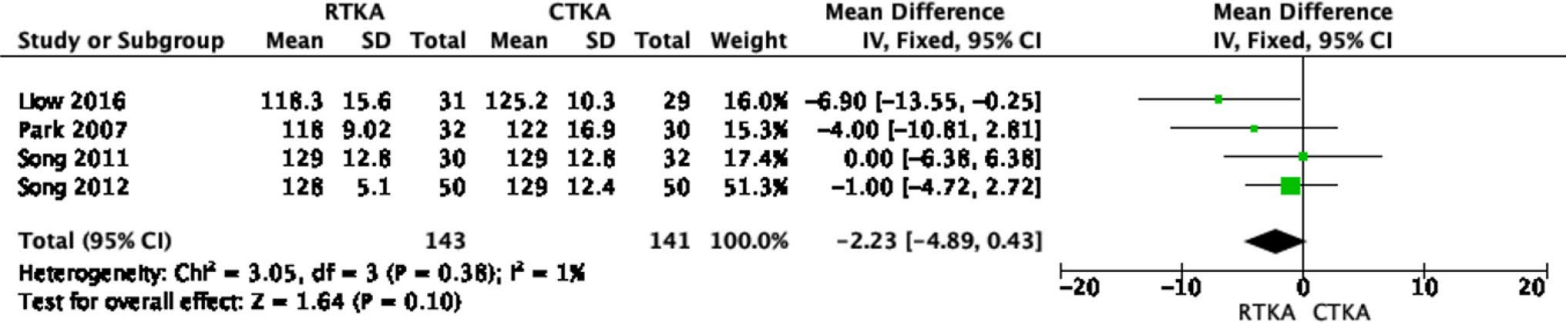
Forest plot of range of motion score at the final follow up between RTKA and CTKA, CI confidence interval

Four studies [19–22] reported on the post-operative range of motion, with their analysis being conducted at the final follow-up, which ranged from 12 to 47 months. The fixed effect meta-analysis indicated a superiority of RTKA knees in terms of range of motion; however, this was not statistically significant, as shown in Fig. 6 (OR − 2.23; 95% CI − 4.89–0.43, *p* value 0.1). The results showed low heterogeneity (*I*^2^ = 1%).

### Complications

**Fig. 7:**
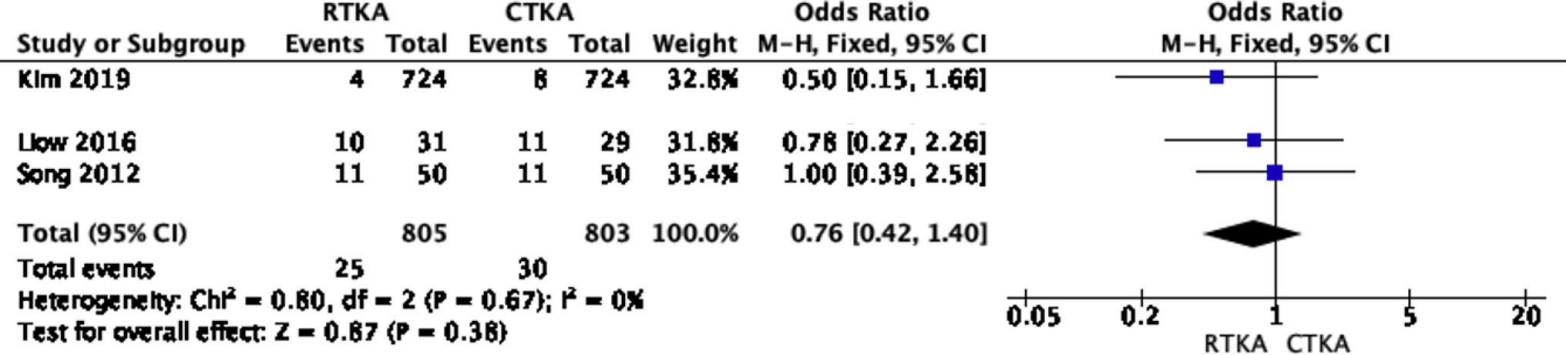
Forest plot of complications at the final follow up between RTKA and CTKA, CI confidence interval

Three studies [18, 19, 22] reported on complication events, with respective follow-up periods ranging from 36 to 120 months. Neither the included studies nor our fixed model analysis at the final follow-up reported any significant differences between RTKA and CTKA. This is displayed in Fig. 7 (OR 0.76; 95% CI, 0.42–1.4, *p* value 0.38). The results showed low heterogeneity (*I*^2^ = 0%). The pooled complications primarily consisted of infection, aseptic loosening, and knee stiffness.

### Post-operative alignment

**Fig. 8:**
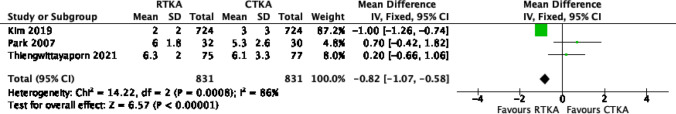
Forest plot of tibiofemoral axis at final follow up between RTKA and CTKA, CI confidence interval

**Fig. 9:**
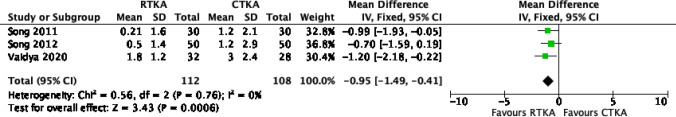
Forest plot of mechanical axis at final follow up between RTKA and CTKA, CI confidence interval

Post-operative alignment parameters were extensively studied in the included papers. The tibiofemoral [18, 20, 23] and mechanical axes [21, 22, 24] were reported in three studies each. As demonstrated in Fig. 8 (OR − 0.82; 95% CI, − 1.027 to − 0.58, *p* value < 0.00001) and Fig. 9 (OR − 0.95; 95% CI, − 1.49 to − 0.41, *p* value < 0.0006), the robotic knee was statistically superior in restoring the tibiofemoral and mechanical axes, respectively. However, the results for the tibiofemoral axis demonstrated high heterogeneity.

**Fig. 10:**
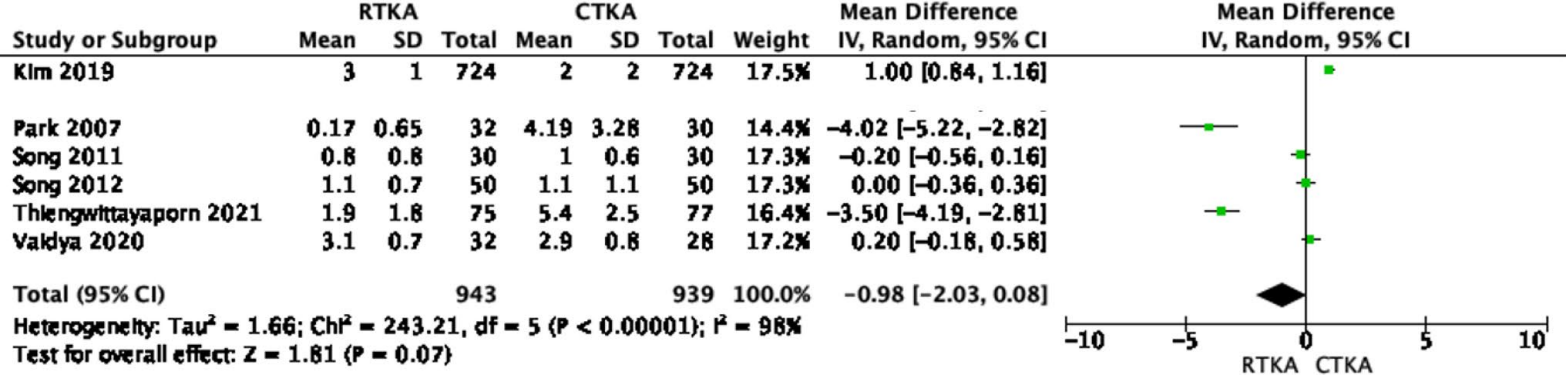
Forest plot of femoral flexion angle at final follow up between RTKA and CTKA, CI confidence interval

**Fig. 11:**
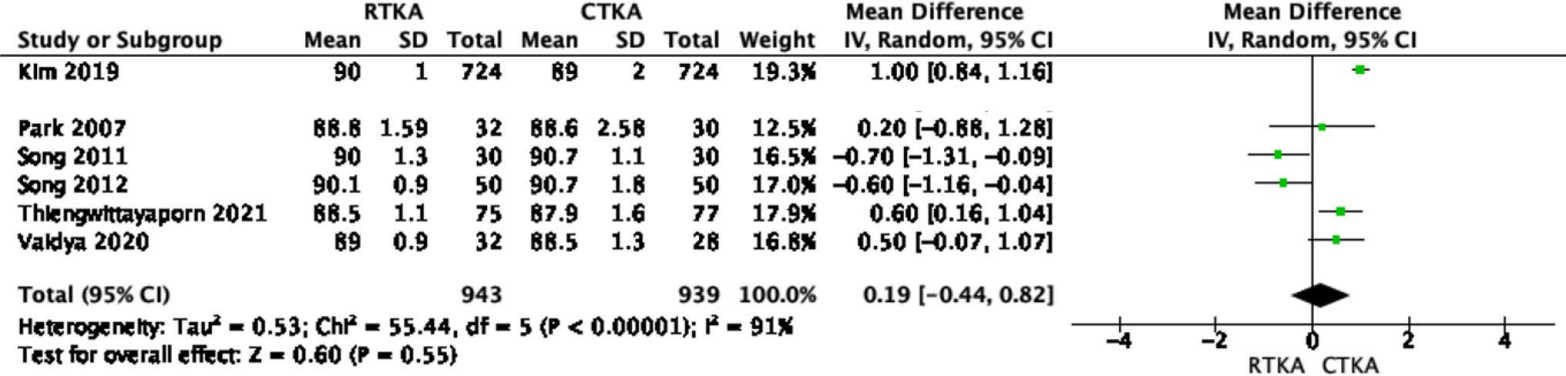
Forest plot of anteroposterior tibial angle at final follow up between RTKA and CTKA, CI confidence interval

**Fig. 12:**
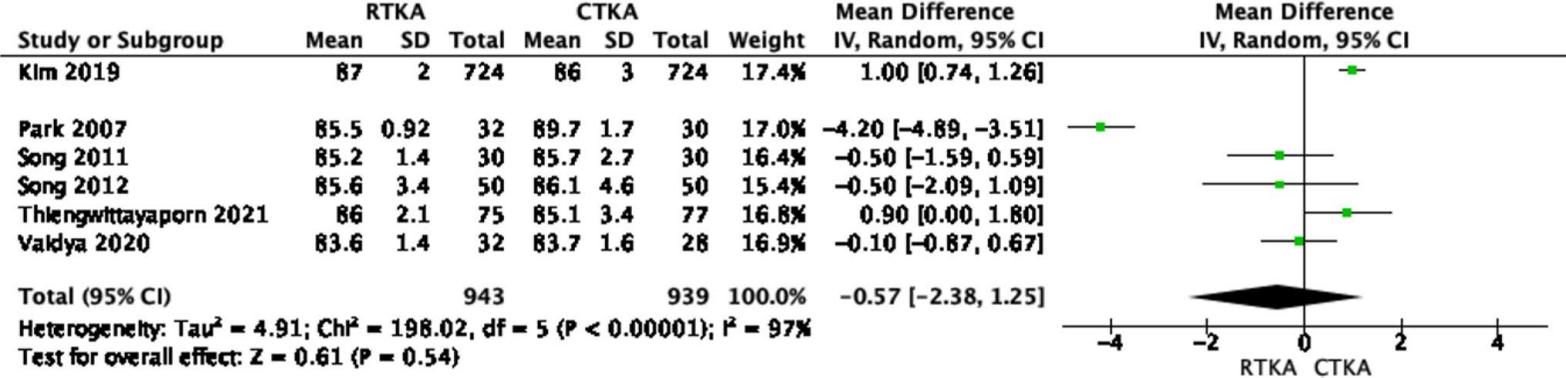
Forest plot of lateral tibial angle at final follow up between RTKA and CTKA, CI confidence interval

On the other hand, other parameters such as the femoral flexion angle, and the anteroposterior and lateral tibial angles were reported in six studies [18, 20–24]. Our fixed model analysis did not reveal any significant differences between the anteroposterior and lateral tibial angles in both knee arthroplasty techniques (*p* value > 0.05), as shown in Figs. 10 and 11. The restoration of the femoral flexion angle favored robotic knees, but this was not statistically significant, as shown in Fig. 12 (OR − 0.98; 95% CI, − 2.03–0.08, *p* value 0.07), with high heterogeneity across the results (*I*^2^ = 98%).

## Discussion

The increase in knee replacement surgeries, mainly due to osteoarthritis, has advanced both conventional and robotic techniques. Despite technological advancements, achieving optimal patient satisfaction remains a challenge, with precise implant placement and soft tissue balance being key factors. RTKA emerged to address these issues, promising enhanced accuracy. This meta-analysis was crucial as it sought to compare the RTKA with CTKA methods.

The most significant result from this meta-analysis was the markedly improved restoration of mechanical alignment in robotic-assisted knee arthroplasty compared to jig-based methods. This finding, which was a unique outcome of our level I study, could likely be credited to the accuracy of knee balancing in robotic surgeries [26, 27]. Furthermore, our study findings concur with prior acknowledgments concerning the superior performance of robotic-assisted knee arthroplasty in terms of implant placement and reduction of radiological outliers.

The precision of robotic knees concerning balancing and implant placement has been shown to positively influence knee outcomes in a few cohorts [7, 28–30]. However, this meta-analysis was unable to demonstrate any superiority in patient-reported outcome scores. Furthermore, the introduction of kinematic alignment, which can be replicated in robotic knees, has increased the popularity of robotic arthroplasty among surgeons [31]. This contrasts with traditional knees that are balanced through measured resection, gap balancing, or a combination of techniques. While kinematic alignment seems to be a promising direction for knee balancing, no long-term studies currently confirm its superiority over other balancing techniques. On the other hand, while RTKA promises theoretical precision, the substantial learning curve and extended operative times present notable concerns. The requirement for preoperative CT imaging with certain systems further adds to the duration, cost, and radiation exposure, complicating its widespread adoption [32–34].

While robotics may yield improved clinical and radiological outcomes, it is necessary to weigh these potential benefits against the increased cost of robotic surgery. The crucial question is whether the possible clinical advantage justifies the incremental cost per knee. Certain robotic systems employ CT scans, which can further escalate costs [28, 35]. Several studies have estimated an increased cost per knee of $1000–1350, and this does not take into account any additional radiological studies that may be required [36, 37].

Several meta-Analyzes in the existing literature compare Robotic Total Knee Arthroplasty (RTKA) to Conventional Total Knee Arthroplasty (CTKA), with none qualifying as a Level I study. For example, Ren et al. [38], in their meta-analysis, which considered 517 knees across 6 RCTs and one cohort study, reported superior restoration of mechanical alignment in RTKA procedures. On the other hand, Onggo et al. [39] reviewed the outcomes of 6500 knees from 18 studies (only 4 RCTs) and intriguingly reported better mechanical alignment restoration, reduced blood loss, and superior clinical outcomes in RTKA surgeries. Additionally, Zhang et al. [40] included 16 studies (no RCT) in their relatively recent meta-analysis, with results favoring RTKA in terms of implant position and early patient-reported outcomes, though the latter was not statistically significant. Our study, in contrast, provides the highest level of evidence on this topic, as it includes the only seven available RCTs in the literature that compare both techniques. The statistical significance reported for post-operative alignment with robotic knee arthroplasty suggests that this method can more accurately achieve the desired alignment compared to conventional methods particularly in mechanical axis and tibiofemoral axis results. However, the presence of heterogeneity in the results for the tibiofemoral axis warrants cautious interpretation. Moreover, the clinical importance of these findings and their impact on patient outcomes and satisfaction remains uncertain. A recent review [41], despite including studies of lower evidence levels, suggests that both neutral (0°–3°) and mild varus (3°–6°) alignments post-TKA result in similar patient outcomes in patients with preoperative varus knees. This highlights the necessity for a more comprehensive exploration of the role alignment plays in the success of TKA.

While this report presented data from studies with high levels of evidence, we acknowledge a few limitations. First, there was variability in the follow-up periods among the included RCTs, and some of the outcomes were analyzed at the final follow-up, which ranged from 2 to 5 years. Second, we were only able to include two robotic systems in our study, one of which, ROBODOC (Curexo Technology, Fremont, CA) [12], is a first-generation robotic system. It is not as widely used currently in comparison to the newer and more advanced robotics. There were no RCTs comparing the use of other robotic systems, such as Mako, which might be more commonly used in certain regions. Lastly, the number of patients involved in the included studies is relatively small, limiting the generalizability of our results. Therefore, further high-powered randomized trials using newer robotic systems are needed to provide a definitive statement about robotic knees.

## Conclusion

This meta-analysis indicates that robotic total knee replacements offer superior post-operative anatomical and mechanical alignment compared to conventional total knee replacements. Despite this, clinical and functional outcomes, as well as complication rates, were similar between the two. These findings should be considered in light of potential confounding factors. More randomized controlled trials with the latest robotic systems are needed to confirm any superior functional and clinical outcomes from robotic-assisted surgeries.

## Data Availability

Not applicable as this is a review article. However, happy to provide access to any statistical data (coding) upon request.
